# A statistical method utilizing information of imported cases to estimate the transmissibility for an influenza pandemic

**DOI:** 10.1186/s12874-017-0300-1

**Published:** 2017-02-21

**Authors:** Ka Chun Chong, Benny Chung Ying Zee, Maggie Haitian Wang

**Affiliations:** 1 Division of Biostatistics, JC School of Public Health and Primary Care, The Chinese University of Hong Kong, Hong Kong, China; 2 Clinical Trials and Biostatistics Laboratory, Shenzhen Research Institute, The Chinese University of Hong Kong, Hong Kong, China

**Keywords:** Basic reproduction number, Epidemic models, Travel data, SIR model, Influenza pandemic

## Abstract

**Background:**

In a new influenza pandemic, travel data such as arrival times of cases seeded by the originating country can be regarded as a combination of the epidemic size and the mobility networks of infections connecting the originating country with other regions. It can be a complete and timely source for estimating the basic reproduction number (*R*_*0*_), a key indicator of disease transmissibility.

**Method:**

In this study, we developed a likelihood-based method using arrival times of infected cases in different countries to estimate *R*_*0*_ for influenza pandemics. A simulation was conducted to assess the performance of the proposed method. We further applied the method to the outbreak of the influenza pandemic A/H1N1 in Mexico.

**Results:**

In the numerical application, the estimated *R*_*0*_ was equal to 1.69 with a 95% confidence interval (1.65, 1.73). For the simulation results, the estimations were robust to the decline of travel rate and other parameter assumptions. Nevertheless, the estimates were moderately sensitive to the assumption of infectious duration. Generally, the findings were in line with other relevant studies.

**Conclusions:**

Our approach as well as the estimate is potential to assist officials in planning control and prevention measures. Improved coordination to streamline or even centralize surveillance of imported cases among countries will thus be beneficial to public health.

## Background

In responding to infectious disease outbreaks in the 21st century, a reliable method to estimate the transmission intensity during the early phases of a new influenza pandemic is critical. The basic reproduction number (*R*_*0*_), defined as the average number of secondary infections produced by a typical infectious individual in a whole susceptible population, is a common measure of disease transmissibility. Valid and reliable estimate of the *R*_*0*_ can assist officials in planning control and prevention measures for an influenza pandemic. On the contrary, underestimation of the disease transmissibility will induce an insufficient public awareness on the risk of infection and therefore lower the general public’s incentive to protect themselves against infectious diseases through vaccines, hand washing or other protective measures.

Many kinds of data such as sentinel surveillance of influenza-like-illness (ILI), serological survey, and syndromic data are able to infer the transmissibility of infectious diseases. Common surveillance data can be fitted into an exponential growth model or Kermack-McKendrick-type models to estimate *R*_*0*_ [1, 2]. Likelihood-based methods are an alternative [3, 4]. Chowell et al. [5] showed that these types of methods are not sensitive on *R*_*0*_ estimation given an acceptable goodness of fit. Nevertheless, one of the caveats of using ILI surveillance data is the underreporting although several approaches were developed to adjust this problem [6].

Serological data is another source for inferring transmissibility of an influenza virus [7, 8]. This kind of data helps detecting subclinical infections and the sampling is not affected by the reporting practices. However, it is comparatively costly and requires laboratory resources. A longer time will be taken to ascertain a disease and thus it cannot provide initial estimate for a pandemic outbreak [9]. Seroprevalence surveys are usually suggested to monitor the level of disease spread after a local outbreak in community [8].

In a recent decade, syndromic data such as web search queries [10] are alternative sources to infer estimates of key epidemiological parameters. Nevertheless, the human behaviors of web searching can be highly affected by intensive media coverage and changes of public’s perceptions on disease severity.

Apart from the abovementioned data sources, travel data related to imported cases could be an alternative information to provide an estimate of transmissibility for a new influenza outbreak. During the initial outbreak of a new influenza pandemic, control measures will be initiated at the border points of entry by various concerned countries. On preventing infected persons from seeding local epidemics, local governments may identify the symptomatic passengers by implementing thermal screening, monitor the suspected cases having a travel history to the originating country and even take quarantine measures [11]. Some travel information such as arrival times of infected cases seeded by the originating country is a combination of the epidemic size and the transport network between the originating country and other at-risk countries. By adapting this information, the transmissibility of an originating country during the initial phase of a pandemic can be back-calculated in order to reduce errors from the underestimation of routine surveillance data [9, 12, 13]. The data about importation events is usually timelier than other available data sources such as seroprevalence data. In the H1N1 pandemic, empirical evidence showed that the flow of airline passenger from Mexico had a significant correlation to the detection of exported cases [14]. Because of intense screening for ILI travelers returning from Mexico, ascertainment of early cases in at-risk countries was timelier and complete compared with routine surveillance in Mexico. Hence, it is believed that the data type is able to provide a reliable estimate on disease transmissibility.

In this paper, we developed a likelihood-based method to estimate the *R*_*0*_ for a new influenza pandemic using the information of imported cases i.e. time of the first introduction of infected individuals in different countries seeded by the originating country. The method was demonstrated to the influenza pandemic A/H1N1 (pH1N1) in mid-March 2009.

## Methods

### Mathematical model

Susceptible-Infectious-Recovered (SIR) model is a common model to describe the dynamic system of the infectious disease [1]. For each time point *t* (*t* = 0, 1, 2, 3…), a closed population is divided into three groups (‘compartments’), namely the susceptible (*S(t)*), the infectious (*I(t)*) and the recovered (*R(t)*) populations. In this compartmental model, susceptible individuals (*S(t)*) are infected at a transmission rate *β*; infectious individuals (*I(t)*) recover at a rate *γ*. Given an exponential assumption, the length of the infectious period is equal to 1/*γ*. Using *S*, *I*, and *R* to represent each compartment, the system dynamics can be described by the differential equations:

1 $$ \begin{array}{l}\frac{dS}{dt}=-\frac{\beta}{N} SI=-\frac{\beta}{N}\left({S}_L+{S}_M\right) I\\ {}\frac{dI}{dt}=\frac{\beta}{N} SI-\gamma I\\ {}\frac{dR}{dt}=\gamma I\end{array} $$

*S*_*L*_ is the susceptible size of local population and *S*_*M*_ is the number of visitors in the originating country. Because *S*_*M*_ is far smaller than *S*_*L*_ i.e. *S*_*L*_ ≫ *S*_*M*_, the number of susceptible individuals is approximately equal to the population *N* i.e. *S*_*L*_ *≈ N* in the initial phase of a new epidemic. We assumed that there is no prior immunity for all subjects. We approximated

2 $$ -\frac{\beta}{N} S I=-\frac{\beta}{N}\left({S}_L+{S}_M\right) I\approx -\frac{\beta}{N}{S}_L I $$

and

3 $$ \frac{dI}{dt}=\frac{\beta}{N} S I-\gamma I\approx \left(\beta -\gamma \right) I $$

In the disease transmission process, the basic reproduction number *R*_*0*_ is a measure of disease transmission intensity. By using the linearization method [2], *R*_*0*_ is always equal to *β/γ* by assuming that the whole population is susceptible at first. Given only one infection at time 0, the prevalence *I(t)* in Eq. (2) is

4 $$ I(t)= I(0) \exp \left[\left(\beta -\gamma \right) t\right]= \exp \left[\left({R}_0-1\right)\gamma t\right] $$

### Distribution of arrival times of infected individuals in countries seeded by originating country

Supposed the passengers counts between the seed country and a particular country *i*-th at day *t* is *m*_*i*_ *(t)* and *d* is the average length of stay the travellers in the seed country i.e. *S*_*M*(*t*)_ = *d*∑_*i*_ *m*_*i*_(*t*), the product (*βd/N)m*_*i*_ *(t)I(t)* is the average number of infected cases arisen in country *i*-th at time *t*. Same daily exposure risk to the domestic cases *I(t)* and uniformity of duration of stay are assumed. By assuming Poisson generations, the probability of importing at least an infected individual (*Y*_*i*_ *)* from the originating country at time *t* is

5 $$ {p}_{i, t}= P\left({Y}_i\ge 1\Big| t,{m}_i(t)\right)=\left(1-\theta \right)\left(1- \exp \left[-\frac{\beta d}{N}{m}_i(t) I(t)\right]\right) $$

where *θ* is the proportion of asymptomatic individuals that cannot be discovered by surveillance measures such as border screening [11, 15]. By substituting *I(t)* obtained from the Eq. (4), the probability *p*_*i*,*t*_ is

6 $$ {p}_{i, t}=\left(1-\theta \right)\left[1- \exp \left[-\frac{\gamma d{R}_0{m}_i(t)}{N} \exp \left[\left({R}_0-1\right)\gamma t\right]\right]\right] $$

The first introduction of an infection that arrives on day *t* in country *i*-th follows a geometric distribution with a time-varying parameter *p*_*i*,*t*_. The probability distribution is

7 $$ {P}_i\left( T= t\right)={p}_{i, t}{\displaystyle {\prod}_{j< t}\left(1-{p}_{i, j}\right)} $$

where *j* = 1, 2, 3…*t*-1. Due to different reporting details for case detections in various countries, the times of arrival are sometimes unavailable and only the days of illness onset will be reported during the early period of pandemic. When the time of arrival is unknown, we have assumed that it follows a multinomial distribution with a probability vector **u** = {*u*_*k*_}, *k* = *k*_*min*_,…-1, 0, 1,…*k*_*max*_ (the minus numbers of days represent previous days with a minimum *k*_*min*_ number of days, and the positive days represent following days with a maximum *k*_*max*_ number of days) as the days from arrival to illness onset can be varied [15]. Given that the distribution is known, the adjusted probability is

8 $$ {q}_i={\displaystyle {\sum}_{k< t}{u}_k{P}_i\left( T= t- k\right)} $$

$$ \mathrm{s}.\mathrm{t}.{\displaystyle \sum {u}_k}=1 $$

### Likelihood formulation

Given fixed parameters of *γ, d*, and *θ*, the likelihood for the arrival times of the first infected individuals for all countries is

9 $$ L\left({R}_0\Big|\mathbf{m},\mathbf{t}\right)={\displaystyle \prod_{i=1}^n{q}_i} $$

where **m** = {*m*_*1*_ *(t), m*_*2*_ *(t), m*_*3*_ *(t), …, m*_*n*_ *(t)*} and **t** = {*t*_*1*_ *, t*_*2*_ *, t*_*3*_ *, …, t*_*n*_} for *n* total number of countries that have reported their importing cases from the originating country. The estimate can be obtained by the maximum likelihood estimation (MLE).

### Simulation testing

The simulation testing aimed to assess the performance of the proposed method by varying the levels of disease transmissibility and travel rates. The simulation tested the robustness of decreasing trend of travels and other parameter assumptions on the estimation. In each setting, 10,000 datasets were simulated for each settings of samples size (*n*) i.e. numbers of countries having imported cases. The population *N* was fixed at 1,000,000 people. The mean, standard deviation (SD), average standard errors of means (SE), and 95% credible intervals (Crl) of an estimate in a simulation were obtained to evaluate the performances.

#### Performance at different levels of reproduction numbers and travel rates

The estimation approach was evaluated at different plausible values of *R*_*0*_ (1.2, 1.7, and 2.2) [16, 17]. We defined the low and high travel volumes by setting the daily rates of travel of each country (**m**) which followed uniform distributions (100, 1000) and (1000, 2000) respectively. The average duration of individual stay was fixed at 3 days. The length of infectious period was set as 3 days and the *θ* was fixed at 30%. Based on these settings, the occurrence of infections at day *t* could be simulated by Eq. (6) assuming a Bernoulli distribution, and the arrival times could be further obtained. By fitting the simulated data, the estimation performances at different levels of *R*_*0*_ and travel rates were assessed.

#### Robustness against a decreasing trend of travel

In a pandemic outbreak, the travel rate could be reduced by time due to the risk perception of publics from mass media and international travel advice [18]. A scenario was designed to test the robustness of constant travelling rate assumption against the decreasing pattern of travels. Using the same scenario settings, we considered a linear decreasing trend of daily travel rates by assuming *m*_*i*_ *(t) = m*_*max*_ *(1-rt),* where *r* is the daily dropping rate and *m*_*max*_ is the passenger rate in the initial day. With reference to the information of pH1N1 pandemic, we assumed *m*_*max*_ during an epidemic period followed a uniform distribution (200, 2500) with fixing *r* to be 0.4% and 0.8%.

#### Sensitivity analysis of parameter assumptions

In order to examine the sensitivity of parameter assumptions, the following distributional assumptions were considered:

- 1/*γ* ~ Gamma distribution with a mean of 3 days and 1 day SD
- *θ* ~ Uniform distribution ranged from 10 to 50%
- *d* ~ Uniform distribution ranged from 1 day to 7 days

The datasets were simulated based on these assumptions. Their impacts on the precision levels were drawn by fixing the values of parameters (1/*γ* = 3 days, *θ* = 30%, *d* = 3 days) in the estimation.

### Numerical study: 2009 influenza pandemic a/H1N1

We applied the estimation approach to the Mexico pH1N1 in mid-March 2009. According to the World Health Organization (WHO), there had been an unexpected increase of influenza-like illness cases shown by the routine influenza surveillance in mid-April, which was not the peak of an influenza season for normal outbreaks to take place, of 2009. Subsequent to a report revealing that two cases of an acute respiratory illness were discovered in two children living in the Southern California of the United States and further confirmed as infections of a new strain of H1N1 virus, additional cases were soon discovered in the country. By the end of April, WHO had further raised the pH1N1 alert to Phase Five, although both the general public and the governments still lacked adequate knowledge on the early stages of pH1N1 at that time. Several years later, there was an estimate of around 284 thousands deaths associated with the 2009 pH1N1 [19].

According to the National Council for Population of Mexico, we fixed the population of Mexico (*N*) as 106,682,518 in 2009 [20]. The start date of the pandemic outbreak was set as March 14, 2009 [21]. The arrival time and illness onset time of infected cases seeded by Mexico, and the number of passengers were retrieved from the studies of Fraser et al. [9], Balcan et al. [12], other articles [22–24] and some news from press [25, 26] with evidence that cases were imported from Mexico. Given the number of passengers from Mexico [9], we assumed the daily rate of passengers: 1. distributed uniformity (*m*_*i*_ *(t) = m*_*i*_) and, 2. linearly decreased (*m*_*i*_ *(t) = m*_*max*_ *(1-rt),* where *r* is the daily dropping rate and *m*_*max*_ is the passenger rate in the initial day with the total number of passengers during the epidemic period held fixed. The dropping rate was assumed to be 40% divided by the length of the study period [18]. For those cases with missing arrival dates, the probability of arrival was adjusted with the illness onset date adapting the information from Sakaguchi et al. [15] in Eq. (8) i.e. **u** = {*u*_*−6*_ *,…, u*_*4*_ } = {0.007,…,0.014}. The information for the estimation is listed in Table 1.

**Table 1:** Travel information and case data during the initial outbreak of 2009 pH1N1

Country	Number of passengers^a^	Arrival dates	Illness onset date	Additional reference^b^
United States	2,474,897	-	March 28	-
Canada	101,313	April 8	April 11	-
El Salvador	15,090	April 19	-	-
Colombia	24,535	-	April 14	[26]
United Kingdom	20,513	April 21	April 24	-
Spain	65,724	April 22	April 25	-
France	61,960	-	April 23	[18]
Costa Rica	16,950	April 25	April 25	-
Argentina	24,609	April 25	April 27	[27]
Cuba	42,802	April 25	-	-
The Netherlands	27,640	April 27	-	-
Germany	35,772	-	April 28	-
Hong Kong	35,706	April 30	April 30	[30]
Italy	12,060	April 29	May 3	[31]
Guatemala	39,460	-	May 1	-

^a^The total number of passengers on flights from Mexico to different countries between March and April 2009

^b^Other supporting information from press and other articles in additional to Fraser et al. [9] and Balcan et al. [12]

In addition, the length of the infectious period was fixed at 3 days and the proportion of asymptomatic infections was fixed at 30% [13]. The average duration of stay of travellers was fixed as 3 days. A sensitivity analysis for the parameters was also conducted. The sensitivity of the estimate was tested by setting the infectious duration equal to 2 days and 4 days, *θ* as 10% and 50%, and *d* as 3 days and 5 days respectively.

The simulation was performed on R 2.15.2 [27] and the code can be provided upon requested.

## Results

### Simulations

**Fig. 1:**
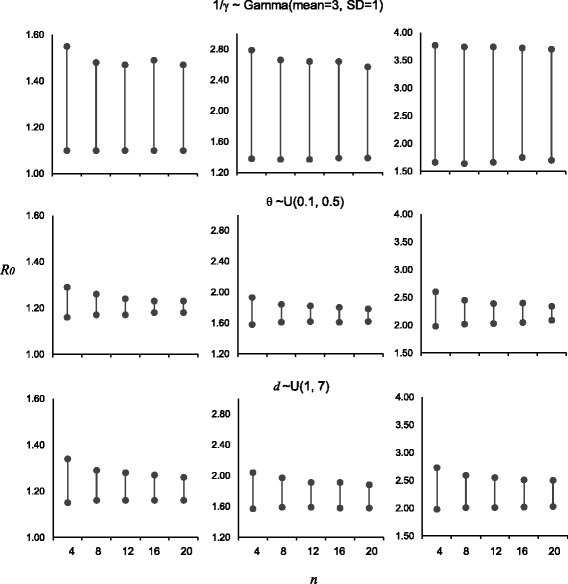
95% credible intervals of the sensitivity analysis for parameter assumptions (1/*γ* ~ Gamma(mean = 3, SD = 1), *θ* ~ U(0.1, 0.5), and *d* ~ U(1, 7) for *top*, *middle*, and *bottom* panels respectively) with true value of *R*_*0*_ equal to 1.2, 1.7, and 2.2 (*left*, *middle*, and *right* columns respectively) in simulations

Table 2 summarizes the estimation performances at different levels of *R*_*0*_ and travel rates. The estimates of the proposed method were close to the true values of different *R*_*0*_ settings. Given *n* = 4, the 95% CrI ranged from 2.08 to 2.43 for an expected *R*_*0*_ of 2.2, whereas the 95% CrI ranged from 1.18 to 1.25 for an expected *R*_*0*_ of 1.2. The results showed that with a low transmissibility, small samples could still achieve a stable precision level, for example, the 95% CrIs were around 1.19 to 1.22 when *n* ≥ 8 in the setting of *R*_*0*_ = 1.2. Higher values of *R*_*0*_ were associated with a slightly lower precision of the estimates. The estimation performance did not show any obvious difference between travel volumes. Apart from that, the estimates from our proposed method were robust to plausible drops of travel rates (Table 3). It was because most cases probably arose in the early phase of pandemic in which changes of travel may not subject to any impact in such a short period of time. The robustness of parameter assumptions was also assessed (Fig. 1). As with other studies, *R*_*0*_ estimation was moderately sensitive to the infectious duration (or the generation interval). Given a plausible distributional assumption, the bias increased and the precision levels decreased with the increase of *R*_*0*_. For example, the estimate was around 2.4 and the 95% CrI ranged from 1.7 to 3.7 for an expected *R*_*0*_ of 2.2. Nevertheless, the estimation performance was not sensitive in the scenarios with lower values of *R*_*0*_. Besides, the simulation results were robust to the assumptions of average stay duration and the proportion of asymptomatic infections. Like most simulation scenarios, the performances were better for a low transmissibility setting.

**Table 2:** Estimation performances at different levels of reproduction numbers and travel rates

		Low travel volumes				High travel volumes			
	*n*	Mean	SD	SE	95% CrI	Mean	SD	SE	95% CrI
*R*_*0*_ = 1.2	4	1.21	0.02	0.01	(1.18, 1.25)	1.21	0.02	0.02	(1.17, 1.25)
	8	1.21	0.01	0.01	(1.18, 1.23)	1.21	0.01	0.01	(1.18, 1.23)
	12	1.21	0.01	0.01	(1.19, 1.22)	1.20	0.01	0.01	(1.18, 1.22)
	16	1.21	0.01	0.01	(1.19, 1.22)	1.20	0.01	0.01	(1.19, 1.22)
	20	1.21	0.01	0.01	(1.19, 1.22)	1.20	0.01	0.01	(1.19, 1.22)
*R*_*0*_ = 1.7	4	1.72	0.05	0.05	(1.64, 1.82)	1.72	0.06	0.05	(1.63, 1.85)
	8	1.71	0.04	0.03	(1.65, 1.79)	1.71	0.04	0.04	(1.64, 1.80)
	12	1.71	0.03	0.03	(1.66, 1.76)	1.71	0.03	0.03	(1.65, 1.78)
	16	1.71	0.02	0.02	(1.66, 1.75)	1.71	0.03	0.03	(1.66, 1.77)
	20	1.70	0.02	0.02	(1.67, 1.74)	1.70	0.02	0.02	(1.66, 1.75)
*R*_*0*_ = 2.2	4	2.22	0.09	0.08	(2.08, 2.43)	2.23	0.11	0.09	(2.04, 2.50)
	8	2.21	0.06	0.06	(2.11, 2.35)	2.21	0.07	0.07	(2.08, 2.38)
	12	2.21	0.05	0.05	(2.11, 2.32)	2.21	0.05	0.05	(2.12, 2.32)
	16	2.21	0.04	0.04	(2.13, 2.29)	2.21	0.05	0.05	(2.12, 2.31)
	20	2.20	0.04	0.04	(2.14, 2.28)	2.21	0.04	0.04	(2.13, 2.31)

*n* numbers of countries having imported cases; *SD* Standard deviation of estimates in a simulation, *SE* Mean standard error of estimates in a simulation, *95% CrI* 95% non-parametric credible intervals

**Table 3:** Robustness of estimates against a decreasing trend of travel rates

		*r* = 0.4%				*r* = 0.8%			
	*n*	Mean	SD	SE	95% CrI	Mean	SD	SE	95% CrI
*R*_*0*_ = 1.2	4	1.20	0.04	0.02	(1.16, 1.30)	1.20	0.04	0.03	(1.14, 1.28)
	8	1.20	0.02	0.02	(1.16, 1.24)	1.20	0.02	0.02	(1.14, 1.25)
	12	1.19	0.01	0.01	(1.17, 1.23)	1.18	0.02	0.01	(1.15, 1.22)
	16	1.19	0.01	0.01	(1.17, 1.22)	1.18	0.01	0.01	(1.15, 1.22)
	20	1.19	0.01	0.01	(1.17, 1.21)	1.18	0.01	0.01	(1.16, 1.21)
*R*_*0*_ = 1.7	4	1.74	0.09	0.07	(1.61, 1.95)	1.75	0.1	0.07	(1.62, 1.97)
	8	1.72	0.06	0.05	(1.62, 1.83)	1.73	0.06	0.05	(1.63, 1.88)
	12	1.72	0.05	0.04	(1.64, 1.82)	1.73	0.05	0.04	(1.65, 1.83)
	16	1.71	0.04	0.04	(1.64, 1.79)	1.72	0.04	0.04	(1.66, 1.81)
	20	1.71	0.04	0.03	(1.66, 1.79)	1.72	0.03	0.03	(1.66, 1.79)
*R*_*0*_ = 2.2	4	2.27	0.14	0.13	(2.03, 2.60)	2.29	0.16	0.13	(2.04, 2.65)
	8	2.25	0.11	0.09	(2.08, 2.49)	2.27	0.1	0.09	(2.10, 2.48)
	12	2.23	0.08	0.07	(2.09, 2.42)	2.25	0.08	0.07	(2.13, 2.42)
	16	2.23	0.07	0.06	(2.11, 2.37)	2.26	0.07	0.06	(2.13, 2.41)
	20	2.23	0.06	0.06	(2.12, 2.36)	2.26	0.06	0.06	(2.15, 2.39)

*n* numbers of countries having imported cases, *r* daily decreasing rate of travels, *SD* Standard deviation of estimates in a simulation, *SE* Mean standard error of estimates in a simulation, *95% CrI* 95% non-parametric credible intervals

### Numerical study

By employing the estimation method, we analyzed the data of the initial outbreak of pH1N1 in Mexico. The estimation results are summarized in Table 4. Showed in the table, the estimation results only have slightly difference in the settings between the constant and linearly decreasing daily rate of passengers. The estimated ${\widehat{R}}_0$ was equal to 1.69 with a 95% confidence intervals (CI) (1.65, 1.73) in a general situation i.e. 1/*γ* = 3 days, *d* = 3 days, and *θ* = 30%. The results were robust to the parameter assumptions (Table 4). Overall the estimated ${\widehat{R}}_0$ s were in range from 1.41 to 2.00 for our tested ranges of parameters. In line with the simulation testing, the effects from *d* and *θ* were comparatively small. The impacts on the estimate were minor for *θ* = 10% (${\widehat{R}}_0=1.67$) and *θ* = 50% (${\widehat{R}}_0=1.73$). Apart from that, the length of infectious period contributed more impact on the ${\widehat{R}}_0$. The estimate ${\widehat{R}}_0$ was equal to 1.45 (95% CI: 1.42, 1.48) when the infectious duration was equal to 2 days, whereas ${\widehat{R}}_0$ was equal to 1.94 (95% CI: 1.88, 1.99) when the infectious duration was equal to 4 days.

**Table 4:** Estimates and 95% confidence intervals (CI) of *R*_*0*_ for the 2009 pH1N1 in Mexico with different lengths of infectious period (1/*γ*), proportions of asymptomatic infections (*θ*), average duration of stays (*d*), and trends of travel rates

			1/*γ* = 3 days		1/*γ* = 2 days		1/*γ* = 4 days	
	Settings		${\widehat{R}}_0$	95% CI	${\widehat{R}}_0$	95% CI	${\widehat{R}}_0$	95% CI
Uniformly travels	*d* = 3 days	*θ* = 30%	1.69	(1.65, 1.73)	1.45	(1.42, 1.48)	1.94	(1.88, 1.99)
		*θ* = 10%	1.67	(1.63, 1.70)	1.44	(1.41, 1.46)	1.91	(1.86, 1.95)
		*θ* = 50%	1.73	(1.69, 1.78)	1.48	(1.44, 1.51)	1.99	(1.93, 2.06)
	*d* = 5 days	*θ* = 30%	1.66	(1.62, 1.70)	1.43	(1.40, 1.45)	1.90	(1.84, 1.95)
		*θ* = 10%	1.64	(1.60, 1.67)	1.41	(1.39, 1.44)	1.86	(1.81, 1.91)
		*θ* = 50%	1.70	(1.65, 1.74)	1.45	(1.42, 1.48)	1.95	(1.88, 2.01)
Decreasing travels	*d* = 3 days	*θ* = 30%	1.70	(1.66, 1.74)	1.46	(1.43, 1.48)	1.95	(1.89, 2.00)
		*θ* = 10%	1.68	(1.64, 1.71)	1.44	(1.41, 1.46)	1.92	(1.87, 1.96)
		*θ* = 50%	1.74	(1.69, 1.79)	1.48	(1.45, 1.51)	2.00	(1.93, 2.06)
	*d* = 5 days	*θ* = 30%	1.68	(1.63, 1.71)	1.43	(1.40, 1.46)	1.90	(1.85, 1.96)
		*θ* = 10%	1.64	(1.60, 1.68)	1.42	(1.39, 1.44)	1.87	(1.82, 1.92)
		*θ* = 50%	1.70	(1.66, 1.75)	1.46	(1.43, 1.49)	1.95	(1.89, 2.01)

## Discussion

Apart from influenza-like-illness, serological survey, and syndromic data, travel data can be an alternative source to timely infer the transmissibility for a new influenza pandemic. Arrival times of cases seeded by the originating country can be regarded as an integration of the number of infected individuals in the originating country and their mobility networks connecting with other regions [28, 29]. By adapting such travel data as a proxy of the size of epidemic expansion, the transmissibility of the influenza virus can be estimated. The estimation based on this information can prevent the bias induced by underestimations of different surveillance data. Undoubtedly, numbers of cases reported by surveillance systems are usually underestimated due to sampling methods, under-detection, and changes of capacities and requirements over time [9, 13, 30]. Apart from that, routine seroprevalence studies that require laboratory resources and a considerably long sample collection time may no longer serve as a suitable monitoring approach during the initial outbreak of a pandemic [31]. In consideration that serological data can refine parameter estimates, a reliable way of using serial cross-sectional serological data alongside with surveillance data to estimate infection rate can be adopted [8]. To address possible errors in the estimation, multi-faceted surveillance measures shall be adopted, especially during the early stages of new influenza outbreaks.

In this study, we developed a likelihood-based approach that employed the arrival times of cases to estimate *R*_*0*_ for a new influenza pandemic. We also conducted a simulation to assess the performance of proposed method. The method was applied to the initial outbreak of influenza pH1N1 in Mexico. We showed that the estimated ${\widehat{R}}_0$ was equal to 1.69 with a 95% CI (1.65, 1.73). The estimated ${\widehat{R}}_0$ and the corresponding range of sensitivity were consistent to other findings of pH1N1, mentioned in the review of *R*_*0*_ from Boëlle et al., the *R*_*0*_s of the 2009 pH1N1 were in range from 1.2 to 2.3 [16]. Compared with other similar studies, Fraser et al. developed a Bayesian method to draw a posterior distribution of *R*_*0*_ for the pH1N1 in Mexico by assuming that tourists infected at a rate proportional to the density of tourists per local resident [12]. The approach adopted the number of infected travelers within a fixed period rather than using the times of exporting infections to countries. Although their estimate (posterior median *R*_*0*_ = 1.4) was similar to us, time to event data combining the mobility and pattern of epidemic invasion is usually preferred to the count data. Balcan et al. used a MLE approach to fit the arrival time data to the simulated epidemics through a highly parameterized meta-population model in which the *R*_*0*_ was their parameter of interest [12]. Their estimate (${\widehat{R}}_0=1.75$) was also closer to our finding. Although their transmission model required many details for parameterization and was computational intensive, it provides an estimate closer to the biological realism over a large population. Nevertheless, we believe our method provides a comparatively simpler way for the transmissibility estimation.

The reliability of the proposed method depends on the quality and quantity of travel data available during the early stage of a influenza pandemic. On estimating the transmissibility, data can be closely aligned with the proposed estimation method when provided with a timely collection of information during the early outbreak. Yet, with this proposed method based on a variety of data sources from the originating country and other regions, differences and incompatibility of surveillance systems across countries, in addition to disparate policies on international reporting and collaboration, have posed challenges to the acquisition of large-scale samples. In pH1N1 of 2009, for instance, not many countries have reported their confirmed cases with known travel history to Mexico [9, 12, 13]. For the betterment of public health in the future, improvements shall be made on the coordination and technical innovation of the streamline or even the centralized surveillance systems for monitoring the imported cases of infections among countries.

One of the advantages of using likelihood-based approach lies in the flexibility in incorporating other available data source. For example, the likelihood can potentially adapt the distribution of days from arrival to illness onset given that the information is available. Moreover, incorporation of demographic data in the transmission model will make the likelihood more informative, as, for instance, younger individuals are more likely to be infected by pH1N1. Yet, the reliability of such extension of the method shall be further justified by sufficient data.

There are several caveats for our study. We did not capture the effects from multi-step journeys in the model although previous articles revealed a single- and multi-step travels did not differ much [32]. Moreover, no adjustment for disease transmissions on aircrafts was incorporated in the estimation. Nevertheless, a retrospective cohort study indicated in-flight transmissions were unlikely [33]. As with many other approaches, we assumed homogeneous mixings at a country level and same risks of infections between travelers and the domestic population. In the early stage of a pandemic, stochastic effects usually induce spatial variations [9]. Apart from that, since airports are the main hubs of connection between countries, passengers will be more likely to be infected in the areas near the airport than areas far from the airports especially for an infectious disease with a short generation interval. Some cases would even recover before their travels. Further developments can take these issues into a consideration.

## Conclusions

The study presents how to use travel data in an influenza pandemic to estimate the basic reproduction number (*R*_*0*_), a key parameter to determine what level of control measures should be used. Compared with other data sources, travel data is relatively more complete and timely for a new pandemic outbreak. Our approach as well as the estimate is potential to assist officials in planning control and prevention measures. Improved coordination as well as centralizing surveillance of imported cases among various regions would thus be beneficial to global health.

## Abbreviations

CI: Confidence interval
CrI: Credible intervals
MLE: Maximum likelihood estimation
pH1N1: Pandemic A/H1N1
SD: Standard deviation
SE: Mean standard error
SIR: Susceptible-Infectious-Recovered model
WHO: World Health Organization
